# Glycemic and Insulinemic Responses of Fresh, Freeze-Dried, and Cooked Apples: As Single Food or Preload

**DOI:** 10.3390/foods14223869

**Published:** 2025-11-12

**Authors:** Jinjie Wei, Anshu Liu, Zhihong Fan, Xiyihe Peng, Xinling Lou, Xuejiao Lu, Jiahui Hu

**Affiliations:** 1 College of Food Science and Nutritional Engineering, China Agricultural University, 17 Qinghua East Road, Beijing 100083, China; 2 Key Laboratory of Precision Nutrition and Food Quality, Department of Nutrition and Health, China Agricultural University, Beijing 100193, China

**Keywords:** apple, freeze-dried fruit, glycemic response, insulin, preload, food matrix

## Abstract

The aim of this study was to investigate the impact of raw, cooking, and freeze-drying on the postprandial glycemic and insulinemic responses and preloading effect of apples. Three fruit products containing 50 g available carbohydrates, including raw apple (RA), freeze-dried apple (FA), and cooked apple (CA), were tested in fourteen healthy subjects. The result showed that the FA elicited both the mildest glycemic and insulinemic responses in terms of early postprandial glucose rise, hypoglycemia risk, insulin peak, and insulin sensitivity among all samples. Compared with CA, the FA induced lower postprandial glycemic amplitude. Compared with its uncooked counterparts, the FA led to lower postprandial insulin excursion. When eaten as a preload prior (containing 15 g available carbohydrates) to a rice meal, all apple preload groups reduced the postprandial glycemic peak, compared with the water preload group. Apple preload enhanced insulin recruitment in the first 30 min after preloading but did not increase the total amount of postprandial insulin secretion. The FA had a higher retention of total phenolic compounds and higher viscosity of the digesta than the RA. This study showed that freeze-dried apples have a potential advantage in terms of glycemic and insulinemic properties. Further investigation on the micro-structure and the rheological properties of the digesta are needed to understand the disparity of glycemic properties between the freeze-dried fruits and the other fruit products.

## 1. Introduction

The health benefits of adequate fruit intake, including improving overall health and reducing the risk of non-communicable diseases, are well proposed by health professionals [1]. Research evidence consistently indicates that the intake of fresh temperate fruits is helpful to diabetes prevention and glycated hemoglobin regulation [2]. In addition to fresh fruit, dried fruit is recognized as valid contributors to daily fruit consumption recommendations [3,4,5].

Epidemiological studies suggest an association between dried fruit consumption and enhanced dietary quality [6]. Clinical investigations demonstrate that regular dried fruit consumption correlates with favorable lipid profiles, improved appetite and satiety regulation, optimized glucose and insulin homeostasis, and reduced risks of cardiovascular diseases and cancer [7,8,9,10,11].

Apple (*Malus pumila* Mill.), one of the most widely consumed fruits globally, is regarded as a healthy food choice beneficial to vascular function, lipid metabolism, inflammation, and hyperglycemia [12,13,14,15,16]. Apple can be consumed fresh, dried, or cooked. Both fresh and air-dried apples exhibit low glycemic index (GI) values, suggesting their potential in postprandial glycemic management when consumed as preloads before high GI carbohydrate meals under isocarbohydrate conditions [17,18]. The canned apple product is also reported as a low GI (42) food [17]. Existing evidence attributes many of apples’ health benefits to their high phenolic and fiber content [19,20]. While the fruit’s fructose and organic acid composition may contribute to its low glycemic properties, these factors remain underexplored [21]. However, the preload effect of freeze-dried apple is yet to be investigated.

Growing evidence indicates that nutrient retention and the bioavailability of phytochemicals of fruits are affected by the processing conditions, including heat treatment and oxygen exposure to varying degrees [22]. Conventional fruit drying methods, such as air-drying, often result in significant nutrient degradation, particularly in vitamins and antioxidant compounds. In contrast, freeze-drying has been increasingly adopted for high-value products to maximize nutritional value [23]. The superior retention of nutrients and phytochemicals in freeze-dried fruit might gain some advantage in terms of glycemic properties.

On the other hand, the texture characteristics could play a key role in the digestive process by modulating the viscosity of digesta and buffering capacity [24,25,26,27]. A compact and resilient food texture generally contributes to a slower release of sugars and reduced accessibility to digestive enzymes, resulting in attenuation of postprandial glycemic response [28,29]. Compared with the elastic and cohesive texture of air-dried apple, the vacuum freeze-dried apple has a relatively looser and softer texture. However, in contrast with the cooked apple, the freeze-dried apple maintains a certain degree of hardness and still keeps part of its natural cell wall structure.

Therefore, it is interesting to investigate whether the freeze-dried apples have a distinct postprandial glycemic response (PGR) and insulinemic response (PIR). However, scant research has been reported involving the PGR and PIR of freeze-dried fruit.

In this study, we take the freeze-dried apple as the study material in comparison to fresh fruits and cooked fruits. We assumed that (1) the pattern of postprandial glycemic and insulinemic responses of freeze-dried fruits were different from those of the fresh fruits and cooked fruits; (2) the difference among the fresh apple, freeze-dried apple, and cooked apple may partly be associated with its texture, buffering capacity, and the viscosity of the digesta.

It is not clear whether, if the special textural feature and the viscosity/buffering capacity could be associated with the postprandial physiological responses of processed and cooked fruits, the freeze-dried apple would manifest different glycemic characteristics.

## 2. Materials and Methods

### 2.1. Materials and Processing

No additives were added to FA. Both the raw and freeze-dried Red fuji apples (*Malus pumila* Mill.) were provided by the Haoxiangni Co., Ltd. (Zhengzhou, China). The apple produce was cultivated in the raw material base in Shandong, China. The cooked fruits were prepared in our laboratory. No additive was added to the apple samples.

The raw apple (FA) was diced into 2 cm cubes (only one side with peel) right before test meals. The freeze-dried apple (FA) was provided in nitrogen-filled bags, diced and freeze-dried. The cooked apple (CA) was prepared by putting 441.9 g apple dices and 180 g water into a domestic pressure cooker (1300 W, 5 L, MY-HT5093, Midea Co., Ltd., Foshan, China) and cooking at 115 °C, 35 kPa for 7 min. The peel of the fruit was preserved, while the core was removed.

### 2.2. Subjects

Healthy young volunteers aged 18–25 years were recruited through advertisements on the university bulletin board and social software online. The recruitment criteria were as follows: (1) Normal weight (BMI between 18.9 and 23.9 kg/m^2^); (2) Regular daily intake of three meals without eating disorders; (3) No weight-gain or weight-loss diets undertaken in the past 3 months; (4) No metabolic diseases; (5) No history of food allergies or intolerances; (6) No smoking, alcohol, or drug dependency; (7) Not participating in competitive sports or high-intensity training. Subjects who met the inclusion criteria were invited to the laboratory to undergo repeated oral glucose tolerance tests (OGTTs) and have their weight, height, BMI, body fat percentage, waist and hip circumferences, and basal metabolic rate measured. Diagnosis was based on OGTT criteria of fasting glucose (below 5.6 mmol/L), peak glucose (below 11.1 mmol/L), and two-hour glucose (below 7.8 mmol/L). Eligible subjects signed informed consent forms.

A statistical power analysis was performed using PASS 2021 software (NCSS, Kaysville, UT, USA). Based on the findings of Lu et al. [18] on air-dried apples, the study set a minimum sample size of ten participants in order to detect differences in the incremental area under the postprandial glucose curve (iAUC) with 80% power and a 5% significance level, and the standard deviation (SD) is lower than 55.15 mmol·min/L. Accounting for an anticipated dropout rate of 30%, recruitment was expanded to 14 participants. The study was conducted at the College of Food Science and Nutrition Engineering at China Agricultural University, in accordance with the Declaration of Helsinki. The research protocol was approved by the China Agricultural University Ethics Committee (Ethics No.: 20220201) and registered with the China Clinical Trial Registry (No.: ChiCTR2200057371).

### 2.3. Test Meal and Study Design

#### 2.3.1. Blood Glucose and Insulin Response of RA, CA, and FA

All test meals contained 50 g of available carbohydrates: (1) raw apple (RA); (2) cooked apple (CA); and (3) freeze-dried apple (FA). The cooked fruit was prepared fresh before consumption and the subjects were instructed to consume both the solid and liquid components. To assess the reliability of the study protocol, the glucose control group underwent two separate tests. The glucose solution was prepared by dissolving 55 g of glucose monohydrate in 250 mL of water at room temperature. The composition of the test meals is detailed in Table 1.

Because of the low water content of FA, 400 mL water was provided along with the fruit sample. The subjects were instructed to chew the dry FA and drink water whenever they felt that water was needed for swallowing the food. They were allowed to leave any amount of water if they could not drink all the 400 mL. They were told not to put the FA into water. After the ingestion of FA, an extra 250 mL water was provided. The subjects could drink the water at any time before lunch. In the case of RA and CA, the 250 g water was provided only after the ingestion of fruit samples.

This study employed a randomized crossover design, in which subjects consumed three test meals on three separate mornings in a computer-generated random order. The test order was randomized with http://www.example.com (accessed on 10 February 2022). The trial was open-labeled because the remarkable differences in shape and texture among three test samples made it difficult to blind the test foods. A minimum three-day interval was maintained between tests to ensure adequate washout. Subjects were instructed to eat the same dinner at the school cafeteria each evening before a test day, and to avoid coffee, alcohol, overeating, staying up late, and strenuous exercise.

On test days, subjects arrived at the laboratory at 07:50 after fasting for 12 h. After resting briefly, two fasting plasma glucose samples were collected: one 10 min before the start of the test (−10 min) and one at the start of the test (0 min). The test formally began at 08:00, requiring subjects to finish eating within 10 min. At 120 min, 200 mL of water at room temperature was provided and had to be consumed before the test concluded. Participants must remain seated throughout and are prohibited from discussing the test meal or any food-related topics.

#### 2.3.2. Blood Glucose and Insulin Measurements of RA, CA, and FA

Postprandial blood samples were obtained via multiple finger-prick blood tests within 180 min. Postprandial blood glucose concentrations (from the second drop of blood) were measured using the glucose oxidase method at −10 min, 0 min (fasting), and then at 15, 30, 45, 60, 90, 120, 150, and 180 min after meal intake. This was carried out using a blood glucose meter (LifeScan Inc., Milpitas, CA, USA). At 0, 15, 30, 45, 60, 90, and 120 min, 150 μL of capillary blood was collected by finger-prick into EDTA K2-coated centrifuge tubes (WanDGL Ltd., Jinan, China) and stored immediately on crushed ice. The blood-containing tubes were then centrifuged at 1000 g for 15 min. Sixty microlitres of the resulting supernatant were transferred to new tubes and stored at −80 °C for subsequent analysis. Plasma insulin concentrations were measured using an ELISA assay kit (JunLB Ltd., Beijing, China). For the insulin assay, blood samples were collected at each time point within 3 min to prevent hemolysis. The plasma was separated promptly after collection and stored at −80 °C without any thawing until analysis. All assays were performed in strict accordance with the manufacturer’s protocols by trained personnel. Each sample was measured in duplicate, with intra- and inter-assay coefficients of variation (CVs) of less than 10% and 15%, respectively.

#### 2.3.3. Blood Glucose and Insulin Responses to Rice Meal with RA, CA, and FA as Preload

All test meals contained 50 g of available carbohydrates: (1) a preload meal of raw apple (15 g available carbohydrates) paired with rice (35 g available carbohydrates) (RA + R); (2) a preload meal of cooked apple (15 g available carbohydrates) paired with rice (35 g available carbohydrates) (CA + R); (3) a freeze-dried apple preload meal (15 g available carbohydrates) with rice (35 g available carbohydrates) (FA + R); and (4) a plain water preload meal (66.1 g raw rice + 132.2 g distilled water) with rice (W + R). The weights of the meals were balanced with water. The carbohydrate component and food weight in test meals are detailed in Table 2. Apple preparation methods are described in Section 2.1. The rice (66.1 g of uncooked rice containing 50 g of acid-forming carbohydrates, plus 132.2 g of distilled water) was cooked for 30 min using the ‘Steaming’ function on an electric rice cooker.

This study employed a randomized crossover design, in which subjects consumed four test meals in a random order on four separate mornings, according to a computer-generated sequence (http://www.example.com). All other requirements aligned with Section 2.3.1.

**Figure 1:**
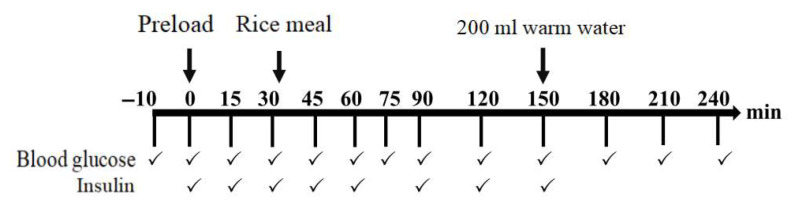
The procedure of trials for the preload study.

The preload test protocol is illustrated in Figure 1. After repeated collection of fasting finger-prick blood samples at −10 and 0 min, the subjects consumed the preload meal within 10 min. The rice meal was provided 30 min after the preload and consumed within 10 min. Blood glucose and insulin levels were measured at fixed time points via finger-prick sampling (as shown in Figure 1), in accordance with the procedure outlined in Section 2.3.2.

### 2.4. Determination of Total Phenolic and Flavonoid Content

Take 7.5 g of fresh, processed fruit samples and homogenize them with 25 mL of 80% methanol for 5 min. Place the mixture in an ultrasonic ice-water bath for 15 min, then centrifuge it at 8000 rpm for 10 min at 4 °C. Perform secondary (10 mL of 80% methanol) and tertiary (5 mL of 80% methanol) extractions on the residue. Collect all the resulting solutions and dilute them to 50 mL with 80% methanol. Each sample was measured in quintuplicate. Total phenolic content (TPC) was determined using the Folin–Ciocalteu microassay described by Jakobek et al. and is expressed as milligrams of gallic acid equivalent (GAE) per 100 g of dry fruit weight (DW) [32]. Total flavonoid content (TFC) was determined using the AlCl_3_ colorimetric method described by Dewanto et al. and expressed as milligrams of catechin equivalent (CE) per 100 g of dry fruit weight (DW) [33]. Moisture content was determined by oven drying. Fruit samples (2000 g) were placed in an oven at 105 °C and dried until a constant weight was reached. The extraction yield for TPC matrix spikes is 84.5 ± 1.5%, while that for TFC matrix spikes is 89.2 ± 3.2%. When sampling fresh apples, cut them into 2 cm cubes, ensuring each cube has only one side with peel.

### 2.5. Texture Analysis

The texture parameters of fresh and processed fruit were determined using a TA.XT Plus texture analyzer (Stable Micro Systems, Godalming, UK), equipped with P/50, P/2, and HDP/BS probes. This was achieved through texture profile analysis (TPA) and puncture and shear tests. For TPA testing, 10 × 10 × 8 mm rectangular cubes of peel-on fruit flesh were cut perpendicular to the peel. The puncture and shear tests used 25 × 25 × 8 mm rectangular cubes that were cut in the same way. The FA was subjected to texture analysis as dry food instead of rehydrated food. Samples were positioned peel-side down during testing. During TPA testing, the probe compressed the sample to a depth of 2.5 mm at a speed of 1 mm/s with a 3 s interval between compressions. Hardness and cohesiveness were recorded [34]. In the puncture test, the probe penetrated the cube to a depth of 5 mm at a speed of 1 mm/s and recorded the puncture force and flexibility (the distance from the trigger point to the break point). In the shear test, the probe vertically sheared the cube to 20 mm at 1 mm/s to obtain shear force and toughness (the incremental area under the force–distance curve) [35]. Each sample was subjected to fifteen replicate measurements.

### 2.6. Buffering Capacity Measurement

The buffering capacity were measured as described by Salaun et al. and Mishra et al. [36,37]. Raw and processed fruit slurry containing 2 g available carbohydrates was dilute with deionized water to 40 g. The homogeneous slurry was each titrated with 0.5 mL of 0.5 M HCl from their initial pH to pH 1.5 followed by titration with 0.5 mL of 0.5 M NaOH to pH 7.0, while the time interval was 1 min. The quantities of acid and alkali used were expressed as mmol, and the difference between acid and alkali used was recorded. The acid buffering capacity (acid BC) was defined as the titratable acidity divided by the total change in pH units (initial pH—1.5) [38]. Each sample was subjected to six replicate measurements.

### 2.7. Viscosity of Fruits After In Vitro Digestion

This study used a three-stage in vitro digestion model to simulate the oral, gastric, and intestinal environments [39]. The fruit was blended with water at a ratio matching that of the test meal (see Table 1) using a blender set to 13,000 rpm for 20 s. Oral phase: Mix 5 g of pulp with 4.975 mL of simulated saliva and 25 μL of 0.3 M CaCl_2_. Heat to 37 °C while shaking at 200 rpm for 2 min. Gastric stage: Add 4 mL of pepsin solution (final enzyme activity = 2000 U/mL), prepared using simulated gastric fluid, and 5 μL of 0.3 mol/L CaCl_2_ to the oral stage product. Adjust the pH to 3.0 using 1 M hydrochloric acid. Incubate the final mixture at 37 °C with shaking for two hours. Intestinal stage: Add 8 mL of pancreatic enzyme solution (prepared to simulate intestinal fluid; final enzyme activity = 100 U/mL based on trypsin activity), 40 μL of 0.3 M CaCl_2_ and 0.6 g of bile (final concentration = 10 mM) to the gastric stage product. Adjust the pH to 7.0 using 1 M NaOH. Shake the final mixture and incubate it at 37 °C for 2 h. After each stage, samples were transferred to −80 °C for freezing. The following day, the samples were thawed overnight at 4 °C in preparation for rheological analysis [40]. Viscosity measurements were performed using a DHR-2 rheometer (TA Instruments, Newark, DE, USA), which was equipped with 40 mm cross-parallel plates and maintained a fixed gap of 1 mm at 37 °C. Each sample was measured three times and equilibrated for 120 s at 37 °C. Steady-state shear flow tests were conducted within the following shear rate ranges: 0.001–50 s^−1^ for oral phase products, and 0.001–30 s^−1^ for gastrointestinal phase products. The behavior of non-Newtonian fluids is typically described using the following power-law function [41]. Each sample was subjected to five replicate measurements.

$$\eta =K\cdot {\dot{\gamma }}^{n-1}$$

While ***η*** represents the apparent viscosity (Pa.s), ***K*** means consistency coefficient (Pa.s^n^), ***γ*** means shear rate (s^−1^), and ***n*** represents flow behavior index.

### 2.8. Statistical Analysis

The fasting blood glucose concentration is calculated as an average of the glucose levels at −10 and 0 min. Postprandial glucose response curves (PGRs) are converted into a measure of glucose elevation relative to fasting values. The following parameters were calculated to extract intrinsic information from PGRs: incremental area under the postprandial glucose curve (iAUC_glu_), incremental area from 0 to 60 min (iAUC_glu0–60_), postprandial glucose peak (Peakglu), standard deviation of glucose fluctuations (SD_glu_), and negative area under the curve (NAUC_glu_). NAUCglu, the area under the postprandial glucose curve, represents the area enclosed by the baseline (i.e., the glucose level at time zero) and the data points below it. Research indicates that the negative area—defined as the portion below the preprandial glucose level that appears after the peak glucose decline—accurately predicts postprandial self-reported hunger and subsequent energy intake. Simultaneously, the PIRs were also converted to the values of insulin rises from the fasting value. The iAUC_ins_, iAUC_ins0–60_, Peak_ins_, and SDins were calculated. Matsuda index ($\frac{10000}{\sqrt{{glucose}_{fasting}*{insulin}_{fasting}*{glucose}_{mean}*{insulin}_{mean}}}$) was calculated [42]. In addition, the postprandial homeostatic model areas under the curve of postprandial insulin × glucose/22.5 (HOMA-IR AUC) were calculated [43].

Repeated-measures linear mixed models were employed to analyze the independent and combined effects of processing methods (raw, cooked, or freeze-dried) and within-subject factors on glycemic and insulin indices [44]. At each time point at which glucose or insulin levels were elevated, the intra-subject variable ‘time’ was added to the model and Pearson’s correlation analysis was used to assess the relationship between GI and II. In addition to GI and II analysis, the model accounted for the following covariates: fasting values, gender, BMI, body fat percentage, basal metabolic rate, waist circumference, hip circumference, and waist-to-hip ratio. All statistical analyses were performed using SPSS version 27.0 (SPSS Inc., Chicago, IL, USA), with *p* < 0.05 being considered statistically significant. Descriptive statistics are presented as mean (standard deviation) unless otherwise specified. Prior to data analysis, the assumptions of the statistical model were verified, including normality and homogeneity of the residual distribution, as well as the linearity of the relationships between the independent and dependent variables.

The physical and chemical parameters of raw and processed fruits were analyzed by one-way ANOVA test or Kruskal–Wallis test, while the viscosity of the fruit digesta were analyzed by general linear models to examine the independent and combined effects of fruit types (RA, CA, FA) and digestion phases (oral, gastric, intestinal). Logarithm or square root transformation of some variables not normally distributed were conducted. Post hoc Tukey adjustments were used to compare means.

## 3. Results

### 3.1. Subjects

**Figure 2:**
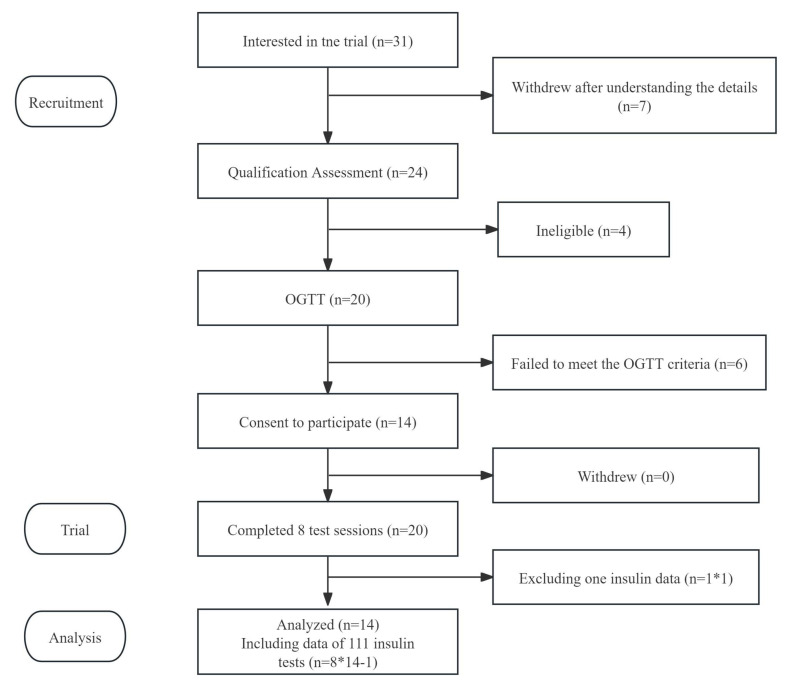
Flow diagram of the study subjects. Notes: “*” means multiplication.

The study subject flow of tests is shown in Figure 2. Fourteen subjects completed all the test sessions, while one insulin test data (AJ) of one participant was excluded due to the hemolysis of the blood samples. The baseline characteristics of subjects are shown in Table 3.

### 3.2. PGRs to Raw and Processed Apple

**Figure 3:**
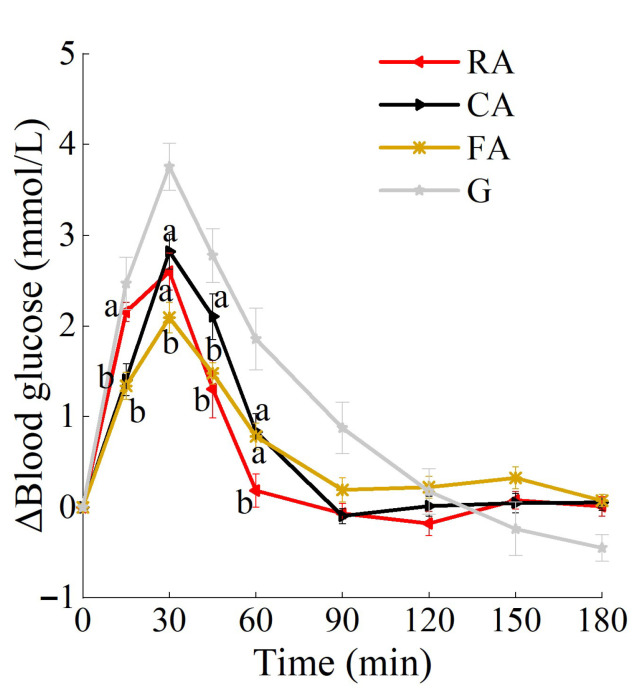
PGRs to raw and processed apples. Treatment time effect, *p* < 0.05. a,b used for comparison between groups at that time point (within the fruit variety, *p* < 0.05) based on repeated-measures linear mixed models with Tukey adjustment. Values are mean ± SD. RA, raw apples; CA, cooked apples; FA, freeze-dried apples.

The changes in postprandial glucose are shown in Figure 3. There was a significant treatment × time effect (*p* < 0.001). All samples reached glucose peaks at 30 min. Compared with both the raw apple and the cooked apple, the freeze-dried apple exhibited a blunted blood glucose response curve.

As shown in Table 4, the treatment effect was significant on iAUC_glu0–60_, NAUC_glu_, Peak_glu_, and SD_glu_. Compared with the RA, the FA induced a significantly more stable PGR characterized by lower NAUC_glu_, Peak_glu_, and SD_glu_. Among the three apple treatments, the RA had the greatest hypoglycemic dip, while the CA caused high glycemic amplitude in the first hour.

### 3.3. PIRs to Raw and Processed Apple

**Figure 4:**
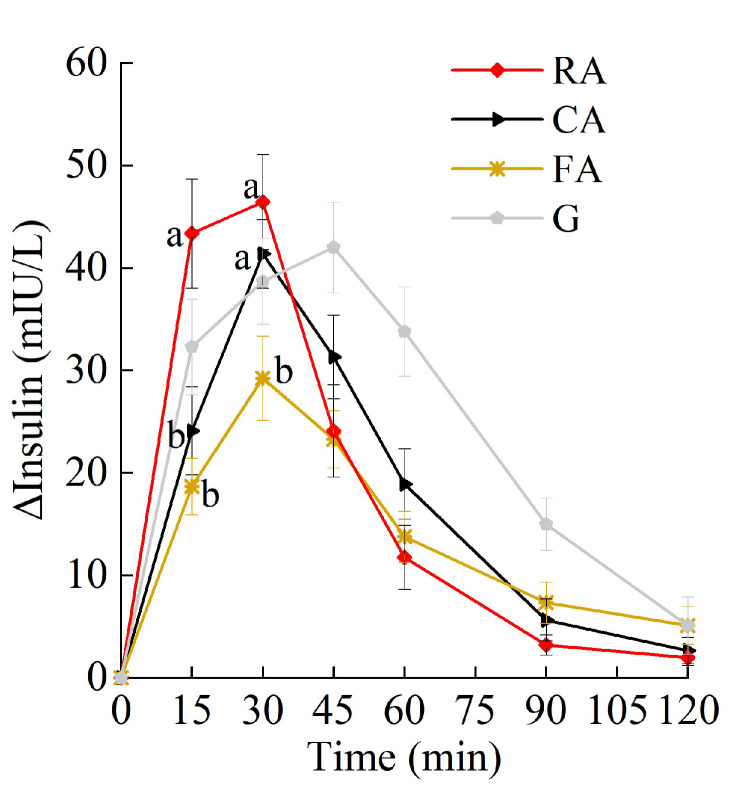
PIRs to raw and processed apples. Treatment time effect, *p* < 0.05. a, b, used for comparison between groups at that time point based on repeated-measures linear mixed models, with Tukey adjustment. Values are mean ± SD. RA, raw apples; CA, cooked apples; FA, freeze-dried apples.

The changes in postprandial insulin are shown in Figure 4. Compared with the RA, the FA induced a smaller insulin increase, exhibiting a significantly lower insulin level at 15 min and 30 min.

As shown in Table 5, there was treatment effect on Peak_ins_, iAUC_ins0–60_, HOMA-IR AUC, SDins, and the insulin index. Compared with RA, the FA led to lower iAUC_ins0–60_, Peak_ins_, and SD_ins_. No significant difference was found in insulin iAUCs between raw and processed apples.

### 3.4. PGRs to Raw and Processed Apple as Preload

**Figure 5:**
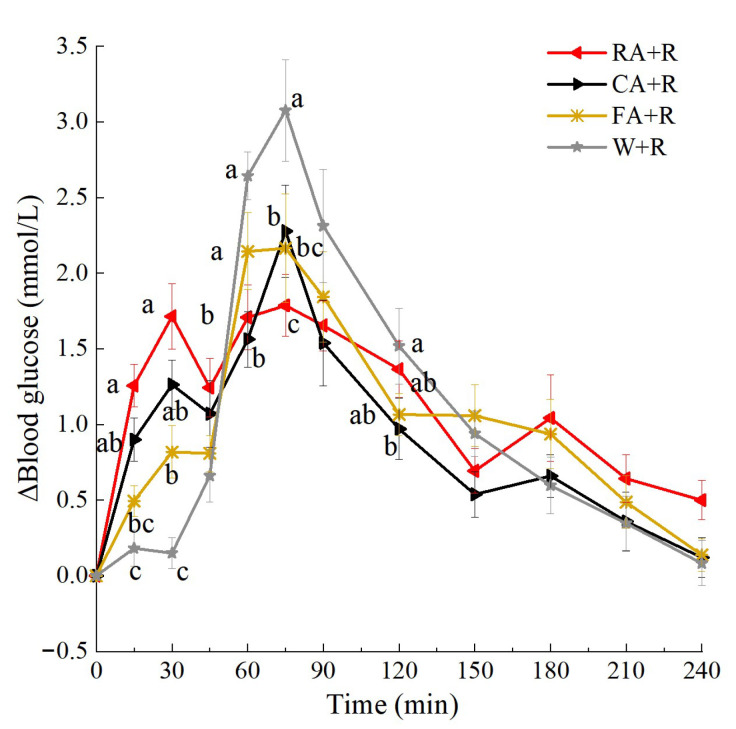
Postprandial glycemic response curves of preload test meals. Treatment effect, *p* < 0.05. a, b, c, used for comparison between groups at that time point (*p* < 0.05) based on repeated-measures linear mixed models with Tukey adjustment. Values are mean ± SD. RA, raw apples; CA, cooked apples; FA, freeze-dried apples.

The glycemic response curves after consumption of the preloads and rice meals are shown in Figure 5. The blood glucose concentrations of all groups peaked at 75 min, i.e., 45 min after the ingestion of rice. At both 15 and 30 min, the RA + R, CA + R, and FA + R groups showed significantly higher blood glucose levels than the W + R group (*p* < 0.05). The blood glucose levels in the RA preload group were significantly higher than those in the FA preload group in the first 30 min (*p* < 0.05). Following rice consumption, the W + R group exhibited significantly higher blood glucose concentrations than both the RA + R and CA + R groups did at 60 min (*p* < 0.05) and significantly exceeded all three apple preload groups at 75 min (*p* < 0.05).

The postprandial glycemic response parameters are listed in Table 6. The iAUC_glu0–30_% was significantly more elevated in the RA + R, CA + R, and FA + R groups than in the W + R control group. Furthermore, the RA + R and CA + R groups demonstrated significantly higher iAUC_glu0–30_% values compared with the FA + R group. Regarding the iAUC_glu30–90_%, both the W + R control and CA + R groups exhibited significantly higher values than the RA + R and FA + R groups. The Peak_glu_ were significantly attenuated in all apple groups (RA + R, CA + R, FA + R) compared with the W + R, with the RA + R group showing the most pronounced reduction (32.3% decrease versus W + R). The CONGA1_glu_ measurements revealed substantial reductions of 42.1%, 42.1%, and 26.3% in the RA + R, CA + R, and FA + R groups, respectively, compared with W + R. Notably, both RA + R and CA + R groups maintained significantly lower CONGA1_glu_ levels than the FA + R group, suggesting enhanced postprandial glycemic stability. SD_glu_ measurements confirmed significantly reduced variability across all treatment groups relative to the water preload group.

**Figure 6:**
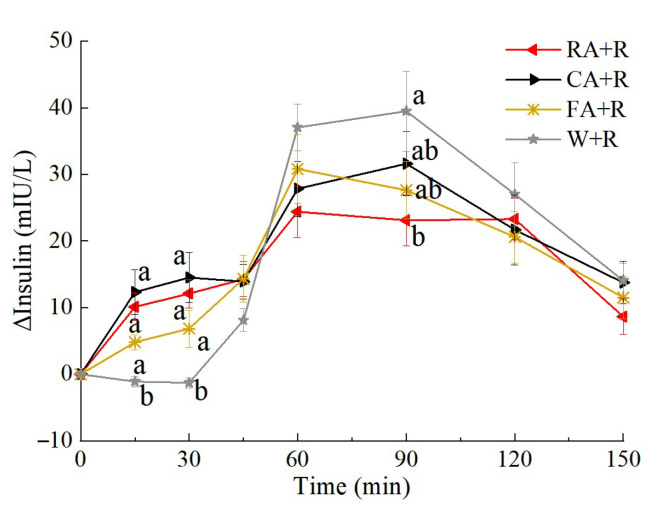
The postprandial insulinemic response curves of the preload test meals. Treatment effect, *p* < 0.05. a, b, used for comparison between groups at that time point based on repeated-measures linear mixed models, with Tukey adjustment. Values are mean ± SD. RA, raw apples; CA, cooked apples; FA, freeze-dried apples.

The insulin response curves resulting from the preload meals are shown in Figure 6. The CA + R and W + R groups exhibited peak insulin concentrations at 90 min postprandially, whereas the RA + R and FA + R groups demonstrated earlier peak responses at 60 min. Comparative analysis showed significantly elevated insulin levels in all fruit preload groups (RA + R, CA + R, FA + R) compared with the water preload control at both 15 min and 30 min. However, this pattern reversed at 90 min, with the W + R group showing significantly higher insulin concentrations than the RA + R group. The FA + R and the RA + R exhibit the highest insulin sensitivity during 0–30 min and 60–90 min, respectively.

The parameters of the postprandial insulin response are shown in Table 7. All fruit preload groups (RA + R, CA + R, FA + R) demonstrated significantly greater iAUC_ins0–30_% compared with the W + R control group (*p* < 0.05), indicating enhanced early-phase insulin secretion following fruit consumption. Notably, the RA + R group exhibited significantly reduced iAUC_ins30–90_% and attenuated Peak_ins_ than all other groups did. Furthermore, while all fruit preload groups showed significantly lower values for both CONIA1_ins_ and SD relative to the control, the RA + R intervention resulted in the most pronounced reduction in CONIA1_ins_ values among the treatment groups.

### 3.5. TPC and TFC

**Figure 7:**
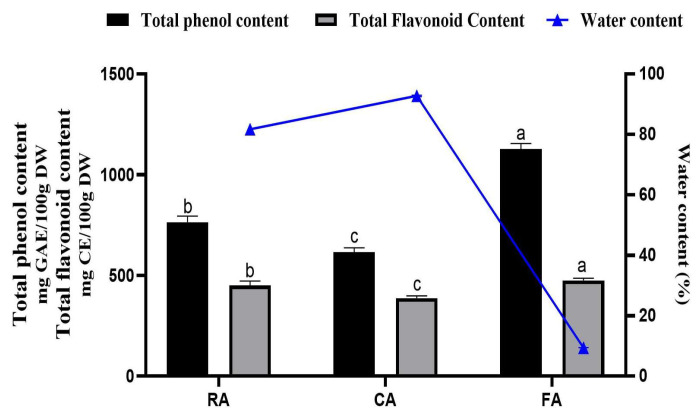
TPC, TFC, and water content of raw and processed fruits (Mean ± SD). a, b, c used for comparison of TPC or TFC between groups based on one-way ANOVA test or Kruskal–Wallis test with Tukey adjustment (*p* < 0.05). RA, raw apples; CA, cooked apples; FA, freeze-dried apples.

The TPC, TFC, and water contents of raw and processed fruits are shown in Figure 7. The freeze-dried fruit possessed the highest phenolic and flavonoid contents. The TPC and TFC decreased significantly after cooking treatments.

### 3.6. Instrumental Texture Parameters

The instrumental texture parameters of raw and processed fruits were shown in Table 8. There were significant differences between groups in all the instrumental texture parameters (*p* < 0.05). After cooking, the hardness, puncture force, shear force, and shear toughness decreased markedly. Compared to RA, FA led to significantly lower cohesiveness, shear force, and shear toughness.

### 3.7. Buffering Capacity

The buffering capacity values of raw and processed apples are shown in Table 9. There was no significant difference in buffering capacity between different treatments.

### 3.8. Viscosity of the Different Digesta

The apparent viscosity decreased as the shear rate increased among all the samples, indicating the shear-thinning behavior.

**Figure 8:**
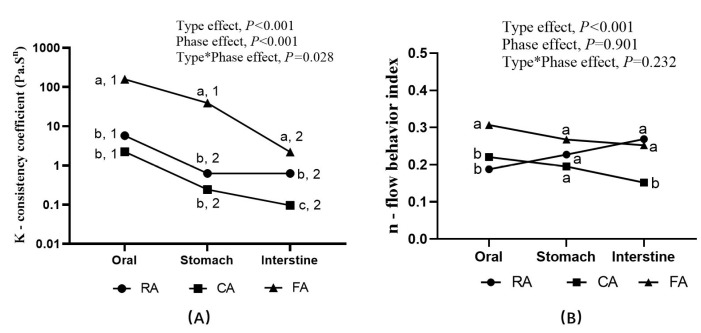
Power-law parameters of the different digesta (Mean). (**A**), consistency coefficient ***K***, type*phase-effect, *p* < 0.001. (**B**), flow behavior index ***n***, type*phase-effect, *p* = 0.232. a, b, c, used for comparison between types (within the phase) based on general linear model with Tukey adjustment (*p* < 0.05). Numbers 1, 2, used for comparison between phases (within the group) based on general linear model with Tukey adjustment (*p* < 0.05). RA, raw apples; CA, cooked apples; FA, freeze-dried apples.

The related power-law parameters are shown in Figure 8. The values of R^2^ were close to 1 (0.90 to 0.99), indicating a good fit of the model to the data points.

The consistency coefficient ***K*** provides information about the consistency of the system. The flow behavior index ***n*** indicates the degree of change in viscosity as the shear rate changes. The lower the value of the flow behavior index, the greater the viscosity decreases with shear rate. The consistency index of all samples, and the difference between raw and processed fruits, decreased along with the digestive process. The viscosity coefficient of freeze-dried apples is higher than that of fresh apples. This may be due to the greater porosity and easier rehydration characteristics of freeze-dried fruit. These properties result in a paste-like consistency that is more viscous upon rehydration [45]. The rheological indices of cooked samples decreased significantly during the small intestine phase in particular, indicating weaker shear stability of the cooked sample bolus.

## 4. Discussion

This study examined the PGRs and PIRs to raw, cooked, and freeze-dried Red Fuji apples, either as the only food of a meal, or as the preload of a rice meal, based on an isocarbohydrate-controlled randomized trial.

In spite of the fact that no significant difference was found in glucose iAUCs among the three apple foods when ingested as the only food in a meal, further analysis of postprandial glycemic markers revealed that freeze-dried apple elicited attenuated postprandial glycemic excursions compared with its raw counterpart. It is impressive that the glycemic and insulinemic patterns of FA can be characterized as a lowered glucose peak, reduced risk of postprandial hypoglycemia, improved glycemic variability, decreased insulin secretion, and elevated insulin sensitivity.

The structural collapse due to moisture removal during dehydration can result in significant changes in food texture. Compared with the fresh apple counterpart, the freeze-dried apple exhibited comparable hardness, puncture force, and puncture flexibility, but greater shear toughness and shear force. Previous studies have indicated that a firm texture and high masticatory effort required for foods can delay the release of sugars from cellular structures, thereby contributing to a lower postprandial glycemic response of FA. However, FA does not exhibit a particularly harder texture compared with the fresh apple. The texture parameters used might be further optimized to identify the difference between the apple samples. Anyway, the present texture parameters cannot explain the disparity between FA and RA. It is acknowledged that the freeze-drying process removes water from apple tissue without disrupting the natural cell wall structure. Like in RA, the release of sugar in RA might be slowed by the dehydrated plant cell structure. Further investigation on the refined micro-structure of the dehydrate fruits is needed to explain the superior insulinemic behavior of the FA.

In addition, since the FA was ingested as dry food, the sugar in the sample might need more time to be released from the dehydrated apple matrix. We provided 400 g drinking water with FA in the test meal, and the subjects were free to drink any amount of water as they felt comfortable to swallow the dried apple meal. However, most of them left some water when they finished the dried apple. In fact, the weights of the three test meals were not precisely balanced: the cooked apple meal was the highest (575 g), followed by the fresh apple meal (442 g), while the freeze-dried apple meal was the lowest (<400 g). We did not match the water intakes among the test meals because it was a real-life situation in which the cooked fruits were ingested with additional water, while the dried fruits were ingested with inadequate water compensation. The reduced amount of fluid intake at the FA meal might further decrease the postprandial glycemic response by modifying the rate of sugar release and gastric emptying. A previous study indicated that liquid intake at a meal or after a meal could accelerate gastric emptying and thus facilitate an elevated postprandial glycemic response [46].

Given the lowered water content, compared with RA, the digesta of FA showed higher apparent viscosity. A highly porous and hydrophilic substrate can lead to a mushy behavior and a more viscous substrate [45]. Accumulated evidence indicated that the viscosity of in vitro digesta had a negative relationship with the postprandial glycemic and insulin responses via delayed gastric emptying, less accessibility of digestive enzymes, and a slowed transportation and absorption of the hydrolyzed nutrients in the gastrointestinal tract [47,48,49]. Hence, the high viscosity of the FA digesta might be one of the factors facilitating slow absorption of sugar, which is conducive to attenuated glycemic and insulin excursion [50,51].

In contrast, compared with the uncooked apple, the CA had much lower values on hardness, shearing toughness, puncture force, and shearing force. The cooking treatment could induce a markedly loosened natural structure and softened texture of fruits, resulting in the release of sugar from apple cells. The looser structure, the softer texture, and the less viscous digesta should have been conducive to an increased glycemic variability and elevated insulinemic response, which was observed in a previous study on cooked air-dried jujube [52].

However, it is interesting that the pressure-cooking treatment did not significantly alter the PGR or PIR of the apple relative to the fresh apple. There is the possibility that the non-texture properties, including the sugar moiety and the presence of organic acid, contribute more to the glycemic and insulinemic characteristics of cooked apple samples.

One of the possible explanations may lie in the sugar composition of the apple. In the test meals, there was about 10 g glucose and 30 g fructose, since the Fuji apple is a rich source of fructose, a low GI sugar. Even when the natural structure of apple was destroyed by heat, the reduced substrate viscosity and accelerated release of 10 g glucose could not support a sustained blood glucose surge.

Another possible explanation might be that the apple contains a considerable amount of organic acid. Due to the increased degree of ionization after dilution, the acidity of the apple did not decrease when cooked in water. It is well accepted that organic acids can stabilize postprandial glycemic response by delaying gastric emptying and attenuating glycemic and insulinemic responses [53,54]. This observation aligns with the findings of Mennah-Govela et al. [55], who established a strong correlation between a food’s initial pH and its buffering capacity, primarily mediated by the ionic composition and organic acid content [56,57]. The combined effect of organic acid content and sugar composition is likely to account for the glycemic behavior of CA.

In the present study, the total phenolics were well preserved in FA, which is consistent with the previous report that the loss of TPC and TFC in freeze-dried fruits was small [58]. Freeze-drying could minimize both the oxidative degradation and thermal decomposition of bioactive compounds, which might contribute to postprandial glycemic regulation through multiple hypothesized mechanisms: inhibition of α-amylase and α-glucosidase enzymatic activity, modulation of intestinal glucose transport, and enhancement of systemic glucose utilization [59,60,61].

However, in the case of CA, the constituents of phenolics might be altered after cooking. On one hand, cooking could induce a certain degree of degradation or oxidation of some phenolic compounds [62]; on the other hand, some phenolic compounds could be hydrolyzed from the cell wall matrix, thus increasing the content of total phenolics [63]. In addition, the loose texture and disrupted cell structure after cooking might enhance both the extraction efficiency in vitro and the bioavailability in the human digestive tract. Therefore, although differences in phenolic content among samples are consistent with the glycemic responses, a causal relationship between the two is yet to be established in the present study.

In the preload trial, all apple preloads attenuated glucose peaks vs. the water control rice meal based on isocarbohydrate consumption, but the differences in glucose and insulin iAUC in 240 min and peak glucose concentration among three apple preloads were modest and time-window dependent. The result suggests that the preload effect of apple on high GI starchy diets [18] might be extended to apple products including cooked and dried apple. However, the low glycemic and low insulinemic properties of FA failed to induce a lower glucose peak compared with FA and CA. This result can be explained by the fact that the FA elicited the least amount of insulin in the 30 min after the meal, while the early recruitment of insulin might be the key to the reduced glucose peak in the apple preload [64].

As far as we know, the current study is the first to report both the postprandial glycemic and insulinemic characteristics and the preload effect of freeze-dried apple.

In the present study, the texture, the buffering capacity, and the viscosity of digesta with glycemic and insulinemic properties in dehydrated and cooked fruit samples were based on the same variety of raw fruit of the same origin, and thus minimized the possible confounders caused by material differences.

With respect to the association between the consumption of processed fruits and health risks, the results in epidemiological studies are consistent on the positive health effect of dried fruits, while inconsistent on other products, including canned fruits. Some found that canned fruits were associated with a better diet quality [65], while some reported negative health effects [55]. The conclusion of these studies might depend on the degree of processing [66]. Our findings indicate that cooked fruits with no added sugar, which can retain considerable amounts of polyphenols and pectin, should not be equated with apple juice or canned fruits, as they may still exhibit medium to low glycemic responses.

This study was focused only on apples instead of multiple fruits. Therefore, the findings in this study should not be simply extrapolated to other fruit varieties. The diversity in glycemic characteristics among fruits following processing and cooking need to be explored.

The association between texture, in vitro digestion indexes, and the PGR/PIR of fruit products is complicated and yet to be further investigated. With respect to the glycemic behavior of dried fruits and cooked fruit products, more future investigation on micro-structures, the oral processing properties, the rate of sugar release from food matrix, and the elicit patterns of incretin after ingestion are needed. The analysis on sugar moiety after cooking is also relevant as dehydration and stewing might induce the degradation of polysaccharides and the formation of new oligosaccharides in the presence of organic acid during heating.

## 5. Conclusions

In conclusion, in our randomized human trials, the freeze-dried apple displayed mild glycemic and insulinemic responses. Compared with the fresh apple, the freeze-dried samples behaved better in terms of early postprandial glucose rise, hypoglycemia risk, insulin peak, and insulin sensitivity. The cooked apple induced a comparable glycemic and insulinemic response with fresh apple. Both the freeze-dried and cooked apple showed remarkable preload effects. The physiological response could partly be associated with the sugar and phenolics content, the texture, the viscosity of digesta, and the buffering features, but the underlying mechanism still needs to be confirmed in future studies.

The favorable glycemic and insulinemic properties of freeze-dried apples suggested their potential of being not only a healthy snack, but also a suitable ingredient for developing glycemic-friendly foods. Though the impact of freeze-drying on glycemic characteristics needs further validation in future studies, the result of the present study warrants further exploration of more freeze-dried fruits to uncover their potential health benefits in glycemic management and in developing new healthy fruit products.

## Figures and Tables

**Table 1:** The ingredients and nutrient composition of test meals (per serving).

	Test Fruits (g)	Water (g) ^a^	AC (g) ^b^		
			Glucose	Fructose	Sucrose
RA ^c^	441.9	250	11.5	30.7	7.4
CA ^c^	575.0	250	11.5	30.7	7.4
FA ^c^	65.0	650	13.4	27.2	9.0

Nutrient content data were acquired through manufacturers and determination experiments according to national standards. (a) According to the international standard for the determination of the GI, the test foods shall be served with 250 g water [30]. (b) Available carbohydrate (AC), which was calculated as follows: 1.05 × (Disaccharides) + Monosaccharides [31]. The sugar moiety of apple sample was determined using ion chromatography. (c) RA, raw apples; CA, cooked apples; FA, freeze-dried apples.

**Table 2:** The ingredients and nutrient composition of test meals (preload) (per serving).

	Preload						Rice Meal	
	Food (g)	Water (g)	AC (g)	Glucose (g)	Sucrose (g)	Fructose (g)	Rice (g) ^a^	AC (g)
RA + R	132.6	23	15	3.5	9.2	2.2	69.9	35
CA + R ^a^	155.6	-	15	3.5	9.2	2.2	69.9	35
FA + R	19.5	136.1	15	3.5	9.2	2.2	69.9	35
W + R	-	54	-	-	-	-	171.5	50

(a) All weights are post-cooking weights.

**Table 3:** Baseline characteristics of participants (*n* = 14).

	Men (*n* = 6)	Women (*n* = 8)
Age, y	24 (2)	22 (1)
BMI, kg/m^2^	22.0 (1.6)	20.9 (1.5)
Fat mass, %	16.6 (3.7)	25.1 (1.5)
Height, cm	175.8 (5.55)	161.3 (5.0)
Weight, kg	68.1 (4.7)	54.6 (5.9)
Waistline, cm	80.5 (4.2)	68.4 (5.5)
Hipline, cm	94.8 (2.4)	91.3 (5.2)
Waist–hip ratio	84.9 (3.9)	74.9 (2.8)
Basal metabolic rate, kcal	161.8 (68.0)	1226.5 (120.8)

**Table 4:** PGRs to raw and processed apples.

Test Meal	iAUC_glu_ (mmol × min/L)	iAUC_glu0–60_%	NAUC_glu_ (mmol × min/L)	Peak_glu_ (mmol/L)	SD_glu_ (mmol/L)
RA	112.5 ± 9.9 ^b^	87.2 ± 4.8 ^a^	22.2 ± 3.5 ^b^	2.8 ± 0.2 ^b^	1.1 ± 0.1 ^b^
CA	125.9 ± 11.0 ^b^	81.0 ± 3.2 ^a^	12.3 ± 3.9 ^b,c^	2.9 ± 0.2 ^b^	1.1 ± 0.1 ^b^
FA	122.4 ± 17.8 ^b^	68.6 ± 2.8 ^b^	7.6 ± 6.0 ^c^	2.1 ± 0.2 ^c^	0.8 ± 0.1 ^c^
G	226.9 ± 21.1 ^a^	69.3 ± 4.493 ^b^	32.4 ± 6.5 ^a^	4.0 ± 0.2 ^a^	1.6 ± 0.2 ^a^

Values are mean ± SD. a–c, used for comparison between groups based on repeated-measures linear mixed models (within fruit variety, *p* < 0.05). RA, raw apples; CA, cooked apples; FA, freeze-dried apples.

**Table 5:** Insulinemic parameters derived from PIRs to raw and processed fruits.

Test Meal	iAUC_ins_ (mIU × min/L)	iAUC_ins0–60_%	Peak_ins_ (mIU/L)	SD_ins_ (mIU/L)	Matsuda Index	HOMA-IR AUC (mmol × mIU\× min/L^2^)
RA	2112.6 ± 235.9 ^b^	86.8 ± 2.4 ^a^	51.4 ± 4.7 ^a^	19.5 ± 1.9 ^a^	121.4± 36.9 ^b^	913.8 ± 97.5 ^b^
CA	2096.2 ± 209.2 ^b^	77.8 ± 4.1 ^b^	44.0 ± 3.7 ^a^	16.3 ± 1.4 ^a^	143.9 ± 46.2 ^b^	920.4 ± 114.5 ^b^
FA	1688.6 ± 205.1 ^b^	71.9 ± 4.1 ^b^	31.2 ± 3.7 ^b^	11.0 ± 1.3 ^b^	142.9 ± 40.7 ^b^	744.5 ± 101.3 ^b^
G	2998.4 ± 208.4 ^a^	65.7 ± 3.1 ^b^	53.6 ± 4.4 ^a^	19.1 ± 1.5 ^a^	161.1 ± 45.7 ^a^	1329.6 ± 120.2 ^a^

Values are mean ± SD. a–b, used for comparison between groups based on repeated-measures linear mixed models (within fruit variety, *p* < 0.05). RA, raw apples; CA, cooked apples; FA, freeze-dried apples.

**Table 6:** The postprandial glycemic parameters of preload test meals.

Test Meal	iAUC_glu240_ (mmol × min/L)	iAUC_glu0–30_%	iAUC_glu30–90_%	Peak_glu_ (mmol/L)	CONGA1_glu_ (mmol/L)	SD_glu_ (mmol/L)
RA + R	275.1 ± 22.3	12.2 ± 1.2 ^a^	37.1 ± 3.6 ^b^	2.3 ± 0.2 ^b^	1.1 ± 0.1 ^c^	0.8 ± 0.1 ^b^
CA + R	224.3 ± 26.9	11.3 ± 1.6 ^a^	44.2 ± 3.6 ^a^	2.5 ± 0.3 ^b^	1.1 ± 0.1 ^c^	0.8 ± 0.1 ^b^
FA + R	250.4 ± 27.8	5.9 ± 1.0 ^b^	39.5 ± 3.1 ^b^	2.6 ± 0.3 ^b^	1.4 ± 0.1 ^b^	0.9 ± 0.1 ^b^
W + R	264.5 ± 32.6	2.1 ± 0.6 ^c^	46.3 ± 2.3 ^a^	3.4 ± 0.3 ^a^	1.9 ± 0.2 ^a^	1.2 ± 0.1 ^a^

Values are mean ± SD. a–c, used for comparison between groups based on repeated-measures linear mixed models (within fruit variety, *p* < 0.05). RA, raw apples; CA, cooked apples; FA, freeze-dried apples.

**Table 7:** The postprandial insulinemic parameters of preload test meals.

Test Meal	iAUC_ins_ (mIU × min/L)	iAUC_ins0–30_%	iAUC_ins30–90_%	Peak_ins_ (mIU/L)	CONIA1_ins_ (mIU/L)	SD_ins_ (mIU/L)
RA + R	2647.0 ± 324.4	9.7 ± 1.4 ^a^	43.4 ± 3.2 ^b^	31.6 ± 3.3 ^b^	16.8 ± 2.7 ^c^	0.8 ± 0.2 ^b^
CA + R	3094.0 ± 416.4	10.0 ± 1.8 ^a^	48.3 ± 3.8 ^a^	40.4 ± 4.5 ^a^	22.6 ± 3.0 ^b^	0.8 ± 0.3 ^b^
FA + R	2732.4 ± 332.9	4.8 ± 0.8 ^b^	49.2 ± 3.4 ^a^	36.6 ± 4.4 ^a^	23.4 ± 4.1 ^b^	0.9 ± 0.3 ^b^
W + R	3228.1 ± 391.2	0.4 ± 0.1 ^c^	50.7 ± 2.8 ^a^	46.8 ± 4.0 ^a^	31.0 ± 2.9 ^a^	1.2 ± 0.3 ^a^

Values are mean ± SD. a–c, used for comparison between groups based on repeated-measures linear mixed models (*p* < 0.05). RA, raw apples; CA, cooked apples; FA, freeze-dried apples.

**Table 8:** Instrumental texture parameters of raw and processed apples (Mean ± SD). a, b, c, used for comparison between groups based on one-way ANOVA test or Kruskal–Wallis test with Tukey adjustment (*p* < 0.05). RA, raw apples; CA, cooked apples; FA, freeze-dried apples.

Test Sample	Hardness (N)	Cohesiveness	Puncture Flexibility (mm)	Puncture Force (N)	Shear Force (g)	Shear Toughness (g × mm)
RA	46.8 ± 5.8 ^a^	0.3 ± 0.02 ^a^	3.7 ± 1.6	3.0 ± 0.3 ^a^	1761.2 ± 159.1 ^a^	4302.6 ± 472.2 ^a^
CA	0.4 ± 0.1 ^b^	0.3 ± 0.03 ^a^	3.5 ± 1.7	0.1 ± 0.01 ^b^	97.6 ± 14.1 ^c^	303.6 ± 86.9 ^c^
FA	42.6 ± 10.0 ^a^	0.1 ± 0.01 ^b^	3.2 ± 1.0	4.4 ± 1.0 ^a^	1018.0 ± 230.6 ^b^	1197.5 ± 212.2 ^b^

**Table 9:** The initial pH and buffering capacity of raw and processed apples. Values are Mean (SD). a used for comparison between groups based on Kruskal–Wallis test with Tukey adjustment (*p* < 0.05). RA, raw apples; CA, cooked apples; FA, freeze-dried apples.

Sample	Initial pH	Acid Used (mmoL)	Alkali Used (mmoL)	Difference (mmoL)	Acid BC (mmoL H+/unit pH)
RA	4.15 (0.08) ^a^	3.00 (0.25) ^a^	3.75 (0.25) ^a^	0.75 (0.25) ^a^	1.13(0.07) ^a^
CA	3.98 (0.03) ^a^	3.00 (0.00) ^a^	3.81 (0.13) ^a^	0.81 (0.13) ^a^	1.21(0.02) ^a^
FA	4.02 (0.01) ^a^	3.00 (0.00) ^a^	3.75 (0.00) ^a^	0.75 (0.00) ^a^	1.20(0.01) ^a^

## Data Availability

The datasets presented in this article are not readily available due to privacy. Requests to access the datasets should be directed to the corresponding author.

## Abbreviations

The following abbreviations are used in this manuscript:

AC	Available carbohydrate
GI	Glycemic index
OGTT	Oral glucose tolerance test
RA + R	Preprandial load of raw apples and rice
CA + R	Preprandial load of cooked apples and rice
FA + R	Preprandial load of freeze-dried apples and rice
W + R	Preprandial load of water and rice
IAUC	Incremental area under the curve
NAUC	Negative area under the curve
CONGA1glu and CONIA1ins	Consecutive 1 h intervals of net glucose/insulin action
PGR	Postprandial glycemic response
PIR	Postprandial insulinemic response
SD	Standard deviation
II	Insulin index
